# A Specific Mixture of Nutrients Suppresses Ovarian Cancer A-2780 Tumor Incidence, Growth, and Metastasis to Lungs

**DOI:** 10.3390/nu9030303

**Published:** 2017-03-18

**Authors:** Mohd Waheed Roomi, Tatiana Kalinovsky, Matthias Rath, Aleksandra Niedzwiecki

**Affiliations:** Dr. Rath Research Institute, Santa Clara, CA 95050, USA; w.roomi @drrath.com (M.W.R.); t.kalinovsky@drrath.com (T.K.); m.rath@drrath.com (M.R.)

**Keywords:** ovarian cancer A-2780 cell line, nutrients, tumor growth, MMPs, Matrigel invasion, metastasis

## Abstract

Ovarian cancer is the deadliest gynecological malignancy in women, and fifth leading cause of death. Despite advances made in chemotherapy and surgery, the average time of clinical remission is approximately 2 years and the 5-year survival rate is 45%. Thus, there is an urgent need for the development of a novel therapeutic approach to ovarian cancer treatment. We investigated the effect of a specific nutrient mixture (EPQ) containing ascorbic acid, lysine, proline, green tea extract, and quercetin on human ovarian cancer cell A-2780 in vivo and in vitro. Athymic female nude mice (*n =* 12) were all inoculated intraperitoneally (IP) with 2 × 10^6^ cells in 0.1 mL of phosphate buffered saline (PBS) and randomly divided into two groups. Upon injection, the Control group (*n =* 6) was fed a regular diet and the EPQ group (*n =* 6) a regular diet supplemented with 0.5% EPQ. Four weeks later, the mice were sacrificed and tumors that developed in the ovary were excised, weighed, and processed for histology. Lungs were inspected for metastasis. In vitro, A-2780 cells were cultured in Dulbecco modified Eagle medium supplemented with 10% FBS and antibiotics. At near confluence, cells were treated with EPQ in triplicate at concentrations between 0 and 1000 μg/mL. Cell proliferation was measured via MTT assay, MMP-9 secretion via gelatinase zymography, invasion through Matrigel and morphology via hematoxylin and eosin (H & E) staining. All Control mice developed large ovarian tumors, whereas 5 out of 6 mice in the EPQ group developed no tumors, and one, a small tumor. Control mice also showed lung metastasis in 6 out of 6 mice, while no lung metastasis was evident in EPQ mice. Zymography demonstrated only MMP-9 expression, which EPQ inhibited in a dose-dependent fashion, with virtual total block at 250 μg/mL concentration. EPQ significantly inhibited invasion through Matrigel with total block at 250 μg/mL concentration. MTT showed dose-dependent inhibition of cell proliferation with EPQ, and H & E staining showed no morphological changes below 500 μg/mL EPQ. These results suggest that EPQ has therapeutic potential in the treatment of ovarian cancer by significantly suppressing ovarian tumor incidence and growth and lung metastasis, and by inhibiting MMP-9 secretion and invasion of A-2780 ovarian cancer cells.

## 1. Introduction

Ovarian carcinoma, which ranks fifth in cancer deaths among women, occurs mainly in post-menopausal women and accounts for more deaths than any other cancer of the female reproductive system [1]. The American Cancer Society estimates that approximately 22,280 women will be diagnosed with ovarian cancer in the United States in 2016 and 14,240 women will die from ovarian cancer [1]. Malignant cancer cells in the ovaries can metastasize directly to other organs in the pelvis and abdomen and through the bloodstream or lymph nodes to other parts of the body [2]. Since ovarian cancer often remains clinically silent, the majority of patients (~60%) are diagnosed with ovarian carcinoma at an advanced stage of intraperitoneal metastatic disease, resulting in a poor prognosis [2]. 

Metastasis occurs secondary to tumor cell adhesion, cell migration, and proteolytic degradation of the extracellular matrix (ECM) by matrix metalloproteinases (MMPs) [3]. MMP-2 and MMP-9 are especially prognostic for survival and metastatic potential in ovarian cancer [4,5,6]. Clinical studies document the association of increased MMP expression with the progression of ovarian cancer [7,8]. Rath and Pauling proposed that nutrients such as lysine and ascorbic acid could act as natural inhibitors of ECM degradation, since these nutrients modulate MMP activity and strengthen the connective tissue surrounding cancer cells, thereby potentially inhibiting tumor growth and progression [9]. We selected naturally occurring nutrients such as lysine, proline, ascorbic acid, green tea extract, and quercetin to target cancer development and developed a nutrient mixture that has exhibited synergistic anticancer activity in vivo and in vitro in a number of cancer cell lines through the inhibition of cancer cell growth, MMP secretion, invasion, metastasis, and angiogenesis [10].

A previous xenograft study on the effect of EPQ on human ovarian cancer ES-2 cell line demonstrated significant inhibition of ES-2 tumor weight and burden by 59.2% (*p <* 0.0001) and 59.7% (*p <* 0.0001), respectively, as well as supportive in vitro results [11]. Though the ES-2 xenograft model was suitable for demonstrating tumorigenicity and testing the effect of therapeutic agents, tumors in this model do not grow in the anatomically correct site, the ovary. Using IP injection of ovarian cancer cells such as A-2780 has shown strong ovarian tumor growth and metastasis, as in human ovarian cancer [12]. Thus, in this study, our objective was to study the effect of supplementation with EPQ on ovarian tumor incidence and growth, and lung metastasis secondary to IP injection of ovarian A-2780 cells in female nude mice, as well as the effect of EPQ in vitro on A-2780 cell proliferation, MMP secretion, and Matrigel invasion. 

## 2. Materials and Methods

Human ovarian cancer cell line A-2780, along with the culture media DMEM, were obtained from ATCC (American Type Culture Collection, Rockville, MD, USA). Antibiotics, penicillin, and fetal bovine serum (FBS) were obtained from Gibco (BRL, Long Island, NY, USA). Twenty-four-well tissue culture plates were obtained from Costar (Cambrdige, MA, USA). Gelatinase zymography was performed in 10% Novex pre-cast SDS polyacrylamide gel (Invitrogen Inc., Carlsbad, CA, USA) with 0.1% gelatin in non-reducing conditions. The nutrient mixture (EPQ), prepared by VitaTech (Hayward, CA, USA) was composed of the following ingredients in the relative amounts indicated: Vitamin C (as ascorbic acid and as Mg, Ca, and palmitate ascorbate) 700 mg; l-lysine 1000 mg; l-proline 750 mg; l-arginine 500 mg; *N*-acetyl cysteine 200 mg; standardized green tea extract (80% polyphenol) 1000 mg; quercetin from quercetin dihydrate, *Saphora japonica* 50 mg; selenium 30 μg; copper 2 mg; manganese 1 mg. All other reagents used were of high quality and were obtained from Sigma, unless otherwise indicated.

### 2.1. In Vivo Studies

#### 2.1.1. Animals

Female nude mice, approximately five weeks of age on arrival, were purchased from Simonsen Laboratories, Gilroy, CA and maintained in microisolator cages under pathogen-free conditions on a 12-h light/12-h dark schedule for a week. All procedures were performed with humane and customary care and use of experimental animals and followed a protocol approved by an internal institutional animal safety review committee.

#### 2.1.2. Experimental Design

After being housed for a week, athymic female nude mice (*n =* 12) were inoculated IP with 2 × 10^6^ cells in 0.1 mL of phosphate buffered saline (PBS). After injection, the mice were randomly divided into two groups; the Control group of mice was fed regular Purina mouse chow and the EPQ group the regular diet supplemented with 0.5% EPQ (*w*/*w*). The regular diet was Laboratory Rodent Diet 5001 from Purina Mills (Gray Summit, MO, USA) LLC/Test Diet. The 0.5% EPQ diet was milled and pressed by Purina Mills, LLC and generated by Vitatech (Tustin, CA, USA). During the study, the mice consumed, on the average, 4 g of their respective diets per day. Thus, the supplemented mice received approximately 20 mg of EPQ per day. Four weeks later, the mice were sacrificed and tumors that developed in the ovary were excised, weighed, and processed for histology.

#### 2.1.3. Histology

Tissue samples were fixed in 10% buffered formalin. All tissues were embedded in paraffin and cut at 4–5 microns. Sections were deparaffinized through xylene and graduated alcohol series to water and stained with hematoxylin and eosin (H & E) for evaluation using a standard light microscope.

### 2.2. In Vitro Studies

#### 2.2.1. Cell Culture

Human ovarian cancer A-2780 cells were grown in DMEM medium supplemented with 10% FBS, penicillin (100 units/mL), and streptomycin (100 μg/mL) in 24-well tissue culture plates. Cells were incubated with 1 mL of media at 37 °C in a tissue culture incubator equilibrated with 95% air and 5% CO_2_. At near confluence, the cells were treated with EPQ, dissolved in media, and tested at 0, 50, 100, 250, 500, and 1000 µg/mL in triplicate at each dose. Phorbol 12-myristate 13-acetate (PMA), 100 ng/mL was added to cells to induce MMP-9 secretion. The plates were then returned to the incubator. 

#### 2.2.2. MTT Assay

Cell viability was evaluated via MTT assay, a colorimetric assay based on the ability of viable cells to reduce a soluble yellow tetrazolium salt (3-(4,5-dimethylthiazol-2-yl) 2,5-diphenyl tetrazolium bromide) (MTT) to a blue formazan crystal by mitochondrial succinate dehydrogenase activity of viable cells. This test is a good index of mitochondrial activity and thus of cell viability. After 24 h of incubation, the cells were washed with PBS and 500 μL of MTT (Sigma #M-2128, St. Louis, MO, USA) 0.5 mg/mL in media was added to each well. After MTT addition (0.5 mg/mL), the plates were covered and returned to the 37 °C incubator for 2 h, the optimal time for formazan product formation. Following incubation, the supernatant was carefully removed from the wells, the formazan product was dissolved in 1 mL of DMSO, and absorbance was measured at 570 nm in Bio Spec 1601, Shimadzu spectrometer (Schimadzu, Santa Clara, CA, USA). The OD_570_ of the DMSO solution in each well was considered to be proportional to the number of cells. The OD_570_ of the control (treatment without supplement) was considered 100%. 

#### 2.2.3. Gelatinase Zymography

Gelatinase zymography was performed in 10% Novex Pre-Cast SDS Polyacrylamide Gel (Invitrogen Corporation, Carlsbad, CA, USA) in the presence of 0.1% gelatin under non-reducing conditions. Culture media (20 μL) were mixed with sample buffer and loaded for SDS-PAGE with tris glycine SDS buffer, as suggested by the manufacturer (Novex). Samples were not boiled before electrophoresis. Following electrophoresis, the gels were washed twice in 2.5% Triton X-100 for 30 min at room temperature to remove SDS. The gels were then incubated at 37 °C overnight in substrate buffer containing 50 mM Tris-HCl and 10 mM CaCl_2_ at pH 8.0 and stained with 0.5% Coomassie Blue R250 in 50% methanol and 10% glacial acetic acid for 30 min and destained. Upon renaturation of the enzyme, the gelatinases digested the gelatin in the gel, producing clear bands against an intensely stained background. Protein standards were run concurrently and approximate molecular weights were determined by plotting the relative mobilities of known proteins. 

#### 2.2.4. Matrigel Invasion

Invasion studies were conducted using Matrigel (Becton Dickinson, San Jose, CA, USA) inserts in 24-well plates. Suspended in medium, A-2780 cells were supplemented with nutrients, as specified in the design of the experiment and seeded on the insert in the well. Thus, both the medium on the insert and in the well contained the same supplements. The plates with the inserts were then incubated in a culture incubator equilibrated with 95% air and 5% CO_2_ for 24 h. After incubation, the media from the wells were withdrawn. The cells on the upper surface of the inserts were gently scrubbed away with cotton swabs. The cells that had penetrated the Matrigel membrane and migrated onto the lower surface of the Matrigel were stained with hematoxylin and eosin and visually counted under the microscope. 

#### 2.2.5. Morphology: H & E

Morphology of cells cultured for 24 h in test concentrations of EPQ were evaluated via H & E staining and observed and photographed by microscopy.

#### 2.2.6. Statistical Analysis

The results were expressed as means ± SD, as indicated in the results, for the groups. Data was analyzed by an independent sample “*t*” test. Pearson’s correlation coefficient and ANOVA one-way variance for the MTT results compared to the concentration of EPQ was run using MedCalc software (Mariakerke, Belgium). IC50 was also analyzed for the MTT results.

## 3. Results

In this study, our objective was to study the effect of supplementation with EPQ on ovarian tumor incidence and growth, and lung metastasis, secondary to IP injection of ovarian A-2780 cells in female nude mice, as well as the effect of EPQ in vitro on A-2780 cell proliferation, MMP secretion, and Matrigel invasion.

### 3.1. In Vivo

#### 3.1.1. Tumor Incidence and Growth

**Figure 1:**
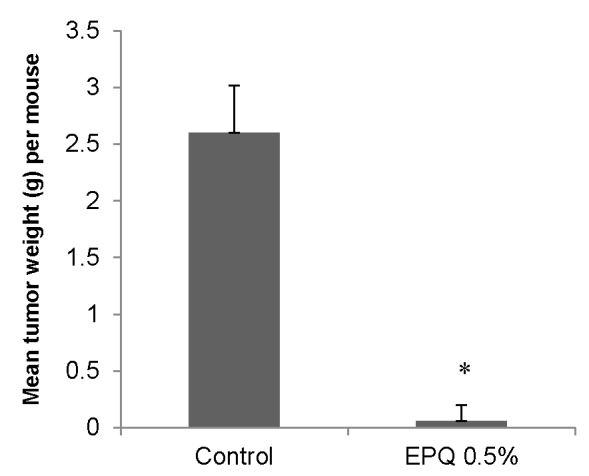
Effect of 0.5% EPQ dietary supplementation on mean weight of A-2780 ovarian tumors in female nude mice injected with 2 × 10^6^ A-2780 cells (* indicates significance of *p <* 0.0001 with respect to control).

**Figure 2:**
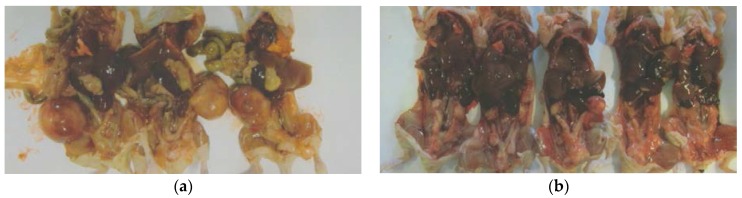
Gross photographs of mice in respective groups. (**a**) Control group, (**b**) EPQ group.

**Figure 3:**
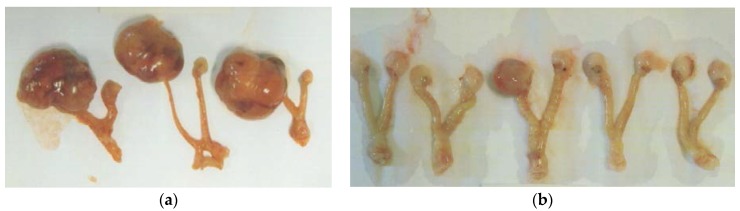
Gross photographs of representative ovarian tumors from groups. (**a**) Control, (**b**) EPQ group.

EPQ strongly inhibited the growth of ovarian tumors in female nude mice. All Control mice developed large ovarian tumors, whereas 5 out of 6 mice in the EPQ group developed no tumors, and one, a small tumor. Mean tumor weight per mouse was inhibited by 98% (*p* < 0.0001) with EPQ 0.5% dietary supplementation, as shown in Figure 1. Mean tumor weight of the Control group was 2.6 ± 0.42 g and mean tumor weight of EPQ group 0.058 ± 0.14 g. See Figure 2 and Figure 3. Mean weight of mice at the initiation of study and that at the termination of study did not differ significantly between groups. 

#### 3.1.2. Tumor Metastasis to Lungs

**Figure 4:**
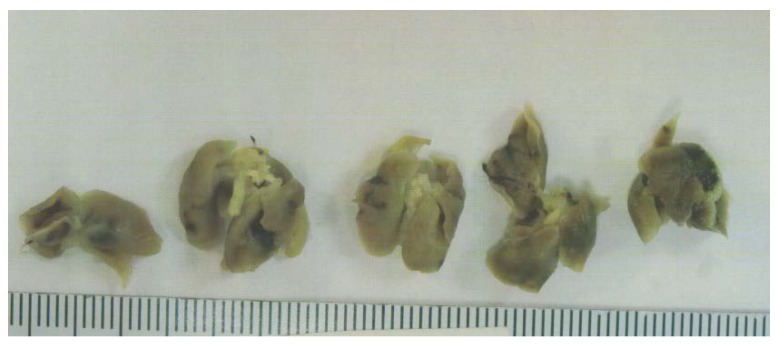
Lung metastasis in Control group: gross photographs.

Tumor metastasis to lungs was found in 6 out of 6 Control mice, whereas no lung metastasis was evident in EPQ mice. See Figure 4.

#### 3.1.3. Histology

**Figure 5:**
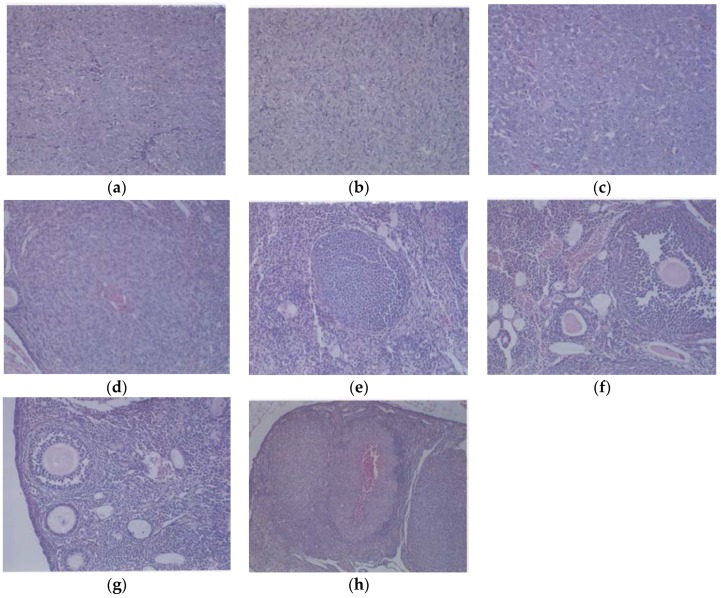
Histology of representative ovaries in groups: magnification 200× except (**h**) which is 100×. Control sections shown in (**a**,**b**). Sections from EPQ-treated mice shown in (**c**–**h**). The representative section shown in (**a**) presents two sections of a large, round tumor from the Control group which appeared to have destroyed the ovary or ovaries. Necrosis varied from 35% in one section to 65% in the second section. Mitotic figures ranged from 0–1 per high-powered field. The section in (**b**) shows a large, round mass from the Control group adjoining a small section of the uterus, suggesting it involves an ovary. Cell morphology was similar to (**a**). Necrosis involved about 50% of the mass. Mitotic figures ranged from 0–1 per high-powered field. The section from the only tumor found in the EPQ group is shown in (**c**). Sections of uterus, cervix, vagina, and ovaries are seen. Mild to moderate multiple small, glandular cysts are present in the uterus. One ovary was totally replaced by a large tumor composed of irregularly, round cells with indistinct cell borders and irregularly round nuclei. Mitotic figures ranged from 4–5 per high-powered field. Multiple foci of necrosis involved about 40% of the tumor mass. The second ovary has two foci of tumor cells. The section shown in (**d**) shows a lesion in the second ovary from the same mouse shown in (**c**) and follicular proliferation is shown in (**e**). In (e), squamous epithelial hyperplasia is present in the cervix and vagina. Multiple, small, glandular cysts are present in the uterus. No overt tumor is present in the ovary. In (**f**), a section of uterus, cervix, vagina, and two ovaries shows multiple developing ovaries present in follicles of both ovaries. In (**g**), representative sections of uterus, cervix, vagina, and two ovaries from mice without ovarian tumors are shown; no significant changes were evident in examined tissues. In (**h**), a section of uterus, cervix, vagina, and two ovaries, multiple, small glandular cysts are present in the uterus. Both ovaries have 1–4 enlarged luteal or follicular structures, composed of sheets of cells morphologically similar to the neoplastic cells in prior tumors.

Histologically, the tumors from both groups were irregularly round and had skin subcutaneous masses, consistent with clear cell ovarian carcinoma; however, the only tumor from the EPQ group was significantly smaller in size than all of the tumors from the Control group. In the Control group, necrosis varied from 35% to 65% and mitotic figures ranged between 0 and 1. See Figure 5a,b for representative sections from the Control group. The EPQ group of mice showed mild to moderate multiple small glandular cysts in the uterus. The only tumor found in the EPQ group, shown in Figure 5c, demonstrated replacement of one ovary with a large tumor composed of irregularly round cells with indistinct cell borders and round nuclei. Necrosis involved 40% of the tumor mass, and mitotic figures ranged from 4–5 per high powered field. Sections shown in Figure 5d,e are from the EPQ mouse with a tumor. Sections from tumor-free EPQ mice are shown in Figure 5f–h. 

### 3.2. In Vitro

#### 3.2.1. Cell Proliferation

**Figure 6:**
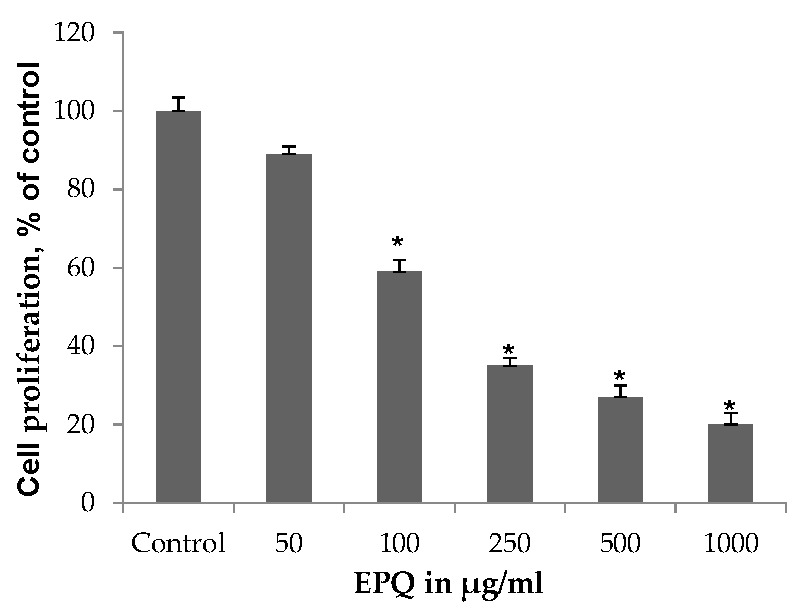
Effect of EPQ on viability of A-2780 cells: MTT 24 h (* indicates significance of at least (*p* ≤ 0.0001) with respect to control). Control solvent is phosphate buffered saline (PBS).

EPQ exhibited significant (*p* ≤ 0.0001, correlation coefficient *r* = −0.834, *p <* 0.0001) dose-dependent inhibition of ovarian A-2780 cell proliferation in vitro with 41%, 65%, 73%, and 80% inhibition of cell growth at 100, 250, 500, and 1000 μg/mL EPQ, respectively, compared to the Control, as shown in Figure 6. ANOVA one-way variance showed a significance level of *p <* 0.001 (F-ratio 362.3, DF 5, 12). IC_50_ was determined to be 120 μg/mL.

#### 3.2.2. Gelatinase Zymography

**Figure 7:**
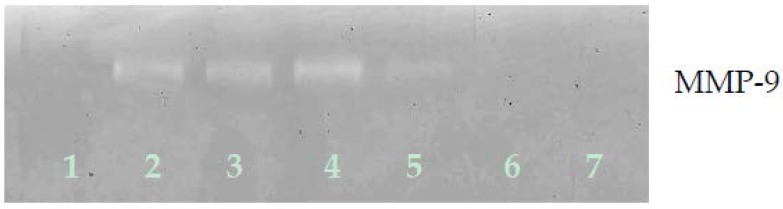
Effect of EPQ on untreated A-2780 cell MMP-2 and MMP-9 secretion. Legend: 1-Control, 2–6 EPQ 50, 100, 250, 500, 1000 μg/mL. Though markers were not clearly seen in the gel image, when running zymography, the band appeared around 92 kDa, which, in our experience, indicates that it is clearly MMP-9.

Zymography demonstrated only MMP-9 expression by ovarian A-2780 cells, which EPQ inhibited in a dose-dependent fashion, with virtual total block at 250 μg/mL concentration, as shown in Figure 7.

#### 3.2.3. Matrigel Invasion

**Figure 8:**
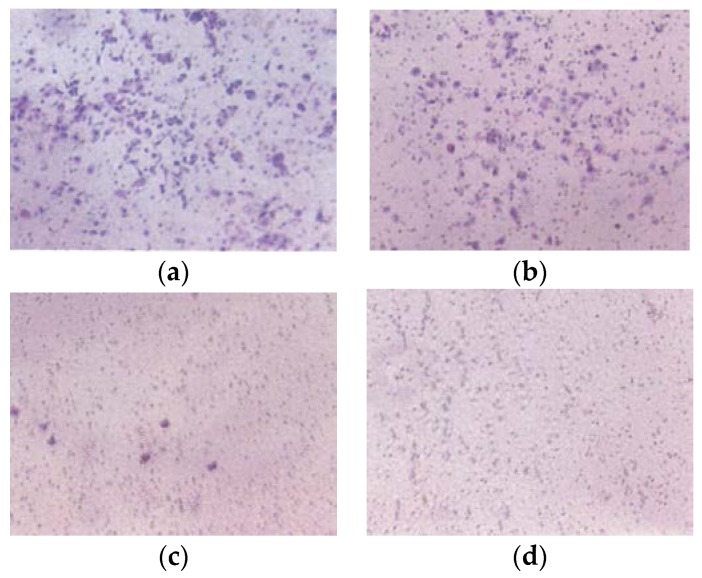
Effect of EPQ on Matrigel invasion of A-2780 cells: photomicrographs (**a**) Control, (**b**) EPQ 50 μg/mL, (**c**) EPQ 100 μg/mL, (**d**) EPQ 250 μg/mL.

EPQ significantly inhibited A-2780 cell invasion through Matrigel in a dose-dependent manner, with total block at 250 μg/mL, as shown in Figure 8.

#### 3.2.4. Morphology: H & E Staining

**Figure 9:**
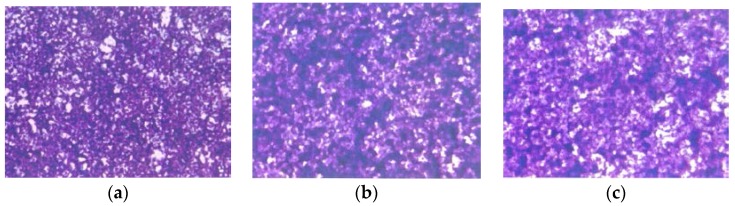

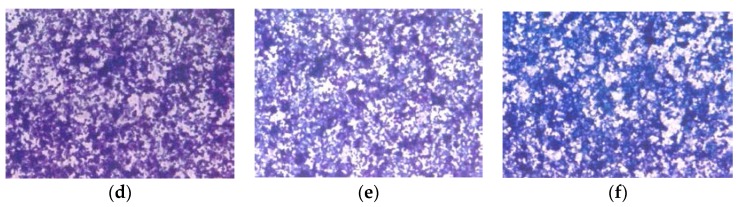
Effect of EPQ on morphology of A-2780 cells: H & E (**a**) Control, (**b**) EPQ 50 μg/mL, (**c**) EPQ 100 μg/mL, (**d**) EPQ 250 μg/mL, (**e**) EPQ 500 μg/mL, (**f**) EPQ 1000 μg/mL.

H &E staining showed no morphological changes below EPQ 100 µg/mL, as shown in Figure 9.

## 4. Discussion

The model of IP injection of ovarian A-2780 cells into female nude mice led to strong ovarian tumor growth and lung metastasis in the control group, as anticipated, thus indicating success of the model. Currently, the cell line utilized is not considered a high-grade serous ovarian cancer cell line; however, A2780 cells do offer the advantage of intraperitoneal growth, which is more realistic than a subcutaneous model. The results of the in vivo study of human ovarian tumor growth in immune impaired (athymic) female nude mice injected IP with A-2780 cells demonstrated significant suppression of ovarian tumor incidence (by 83%) and growth (87%; *p* < 0.0001) with EPQ 0.5% dietary supplementation. Furthermore, EPQ totally inhibited metastasis to lungs compared to Control mice, which exhibited 100% lung metastasis. Serum levels of EPQ in mice were not determined. Results from the in vitro studies support the in vivo findings, as EPQ showed inhibition of MMP-9 secretion with virtual total block at 250 μg/mL EPQ and 100% inhibition of invasion of cells through Matrigel at 250 µg/mL. In addition, EPQ showed increased toxicity to A-2780 cells in a dose-dependent manner, with 80% inhibition of cell growth in cells exposed to 1000 μg/mL EPQ.

High consumption of fruits and vegetables, have been reported to be associated with prevention, inhibition, and reversal of cancers [13,14]. EPQ formulation was based on addressing critical physiological targets in cancer progression and metastasis. Adequate supplies of ascorbic acid, lysine, and proline are essential to ensuring an optimal ECM structure, and lysine supports ECM stability as a natural inhibitor of plasmin-induced proteolysis [9,15]. Collagen formation is also dependent upon manganese and copper. Green tea extract has been shown to be a potent inhibitor of cancer cell growth, metastasis, angiogenesis, and other aspects of cancer progression [16,17,18,19,20,21,22]. Quercetin’s anticancer effects include the induction of cell cycle arrest, apoptosis (without affecting normal cells), and antioxidant activity in vivo and in vitro [23]. Ascorbic acid has been documented to effect cytotoxic and antimetastatic actions on malignant cell lines [24,25,26,27,28,29] and low ascorbate levels are noted in cancer patients [30,31]. Selenium and N-acetyl cysteine demonstrated the inhibition of MMP-9, migration, and invasive activities [32,33,34]. A precursor of nitric oxide, arginine, induces apoptosis [35]. 

## 5. Conclusions

Since current treatment methods for ovarian cancer are generally ineffective, there is a need for the development of effective therapeutic agents for these cancers with minimal toxicity. Our studies demonstrated that the mixture of the non-toxic components of EPQ significantly suppressed ovarian tumor incidence and growth and completely blocked metastasis to lungs. In vitro, MMP-9 secretion and Matrigel invasion were strongly inhibited and EPQ showed potent dose-dependent toxicity to A-2780 cells. These findings suggest a potential of EPQ in the treatment of ovarian cancer.

## References

[1] American Cancer Society: What Are the Key Statistics about Ovarian Cancer?. http://www.cancer.org/cancer/ovariancancer/detailedguide/ovarian-cancer-key-statistics.

[2] Ovarian Cancer National Alliance: Ovarian Cancer: Statistics. http://www.ovariancancer.org/about/statistics/.

[3] Duffy M.J. (1992). The role of proteolytic enzymes in cancer invasion and metastasis. Clin. Exp. Metastasis..

[4] Davidson B., Goldberg I., Gotlieb W.H., Kopoiovic J., Ben-Baruch G., Nesland J.M., Berner A., Byrne M., Reich R. (1999). High levels of MMP-2, MMP-9, MT1-MMP and TIMP-2 mRNA correlate with poor survival in ovarian carcinoma. Clin. Exp. Metastasis.

[5] Davidson B., Goldberg I., Gotlieb W.H., Kopoiovic J., Ben-Baruch G., Nesland J.M., Reich R. (2002). The prognostic value of metalloproteinases and angiogenic factors in ovarian carcinoma. Mol. Cell Endocrinol..

[6] Wu X., Li H., Kang L., Li L., Wang W., Shan B. (2002). Activated matrix metalloproteinase-2 a potential maker of prognosis for epithelial ovarian cancer. Gynecol. Oncol..

[7] Lopata A., Agresta F., Quinn M.A., Smith C., Ostor A.G., Salamonsen L.A. (2003). Detection of endometrial cancer by determination of matrix metalloproteinases in the uterine cavity. Gynecol. Oncol..

[8] Torng P.L., Mao T.L., Chan W.Y., Huang S.C., Lin C.T. (2004). Prognostic significance of stromal metalloproteinase-2 in ovarian adenocarcinoma and in relation to carcinoma progression. Gynecol. Oncol..

[9] Rath M., Pauling L. (1992). Plasmin-induced proteolysis and the role of apoprotein(a), lysine and synthetic analogs. Orthomol. Med..

[10] Niedzwiecki A., Roomi M.W., Kalinovsky T., Rath M. (2010). Micronutrient synergy—A new tool in effective control of metastasis and other key mechanisms of cancer. Cancer Metastasis Rev..

[11] Roomi M.W., Kalinovsky T., Rath M., Niedzwiecki A. (2016). A nutrient mixture modulates ovarian ES-2 cancer progression by inhibiting xenograft tumor growth and cellular MMP secretion, migration and invasion. Int. J. Clin. Exp. Med..

[12] Shaw T.J., Senterman M.K., Dawson K., Crane C.A., Vanderhyden B.C. (2004). Characterization of intraperitoneal, orthotopic and metastatic xenograft models of human ovarian cancer. Mol. Therapy.

[13] Aldercreutz H. (1990). Western diet and Western disease: Some hormonal and biochemical mechanisms and associations. Scand. J. Clin. Lab. Investig..

[14] Miller A.B. (1990). Diet and Cancer: A review. Acta Oncol..

[15] Sun Z., Chen Y.H., Wang P., Zhang J., Gurewich V., Zhang P., Liu J.N. (2002). The blockage of high-affinity lysine binding sites of plasminogen by EACA significantly inhibits prourokinase-induced plasminogen activation. Biochem. Biophys. Acta.

[16] Kemberling J.K., Hampton J.A., Keck R.W., Gomez M.A., Selman S.H. (2003). Inhibition of bladder tumor growth by the green tea derivative epigallocatechin-3-gallate. J. Urol..

[17] Sato D., Matsushima M. (2003). Preventive effects of urinary bladder tumors induced by *N*-butyl-*N*-(4-hydroxybutyl)-nitrosamine in rat by green tea leaves. Int. J. Urol..

[18] Valcic S., Timmermann B.N., Alberts D.S., Wachter G.A., Krutzsch M., Wymer J., Guillen J.M. (1996). Inhibitory effect of six green tea catechins and caffeine on the growth of four selected human tumor cell lines. Anticancer Drugs.

[19] Mukhtar H., Ahmed N. (2000). Tea polyphenols: Prevention of cancer and optimizing health. Am. J. Clin. Nutr..

[20] Yang G.Y., Liao J., Kim K., Yurtow E.J., Yang C.S. (1998). Inhibition of growth and induction of apoptosis in human cancer cell lines by tea polyphenols. Carcinogenesis.

[21] Taniguchi S., Fujiki H., Kobayashi H., Go H., Miyado K., Sadano H., Shimikawa R. (1992). Effect of (-) epigallocatechin gallate, the main constituent of green tea, on lung metastasis with mouse B16 melanoma cell lines. Cancer Lett..

[22] Hara Y. (2001). Green tea: Health Benefits and Applications.

[23] Gibellini L., Pinti M., Nasi M., Montagna J.P., De Blasi S., Roat E., Bertoncelli L., Cooper E.I., Cossarizza A. (2011). Quercetin and cancer chemoprevention. Evid.-Based Complement. Altern. Med..

[24] Cha J., Roomi M.W., Ivanov V., Kalinovsky T., Niedzwiecki A., Rath M. (2013). Ascorbate supplementation inhibits growth and metastasis of B16FO melanoma and 4T1 breast cancer cells in vitamin C-deficient mice. Int. J. Oncol..

[25] Naidu K.A., Karl R.C., Coppola D. (2003). Antiproliferative and proapoptotic effect of ascorbyl stearate in human pancreatic cancer cells: Association with decreased expression of insulin-like growth factor 1 receptor. Dig. Dis. Sci..

[26] Anthony H.M., Schorah C.J. (1982). Severe hypovitaminosis C in lung-cancer patients: The utilization of vitamin C in surgical repair and lymphocyte-related host resistance. Br. J. Cancer.

[27] Maramag C., Menon M., Balaji K.C., Reddy P.G., Laxmanan S. (1997). Effect of vitamin C on prostate cancer cells in vitro: Effect on cell number, viability and DNA synthesis. Prostate.

[28] Koh W.S., Lee S.J., Lee H., Park C., Park M.H., Kim W.S., Yoon S.S., Park K., Hong S.I., Chung M.H., Park C.H. (1998). Differential effects and transport kinetics of ascorbate derivatives in leukemic cell lines. Anticancer Res..

[29] Chen Q., Espey M.G., Krishna M.C., Mitchell J.B., Corpe C.P., Buettner G.R., Shacter E., Levine M. (2005). Pharmacologic ascorbic acid concentrations selectively kill cancer cells: Action as a pro-drug to deliver hydrogen peroxide to tissues. PNAS.

[30] Nunez C., Ortiz de Apodaca Y., Ruiz A. (1995). Ascorbic acid in the plasma and blood cells of women with breast cancer. The effect of consumption of food with an elevated content of this vitamin. Nutr. Hosp..

[31] Kurbacher C.M., Wagner U., Kolster B., Andreotti P.E., Krebs D., Bruckner H.W. (1996). Ascorbic acid (vitamin C) improves the antineoplastic activity of doxorubicin, cisplatin and paclitaxel in human breast carcinoma cells in vitro. Cancer Lett..

[32] Kawakami S., Kageyama Y., Fujii Y., Kihara K., Oshima H. (2001). Inhibitory effects of *N*-acetyl cysteine on invasion and MMP 9 production of T24 human bladder cancer cells. Anticancer Res..

[33] Morini M., Cai T., Aluigi M.G., Noonan D.M., Masiello L., De Floro S., D’Agostinin F., Albini A., Fassima G. (1999). The role of the thiol *N*-acetyl cysteine in the prevention of tumor invasion and angiogenesis. Int. J. Biol. Markers.

[34] Yoon S.O., Kim M.M., Chung A.S. (2001). Inhibitory effects of selenite on invasion of HT 1080 tumor cells. J. Biol. Chem..

[35] Cooke J.P., Dzau V.J. (1997). Nitric oxide synthase: Role in the genesis of vascular disease. Annu. Rev. Med..

